# Hydrological modeling of flood impacts under land use and land cover change: A systematic review of tools, trends, and challenges

**DOI:** 10.1016/j.mex.2025.103724

**Published:** 2025-11-13

**Authors:** Tin Zar Oo, Usa Wannasingha Humphries

**Affiliations:** a The Joint Graduate School of Energy and Environment (JGSEE), King Mongkut's University of Technology Thonburi (KMUTT), Bangkok, 10140, Thailand; b Department of Mathematics, Faculty of Science, King Mongkut’s University of Technology, Thonburi (KMUTT), 126 Pracha Uthit Road, Bang Mod, Thung Khru, Bangkok 10140, Thailand

**Keywords:** Land use and land cover change, Flood dynamics, Hydrological models, PRISMA guidelines, Bibliometric analysis

## Abstract

Land use and land cover (LULC) change is a major anthropogenic factor influencing flood behavior and hydrological processes. This systematic review synthesizes two decades (2005–2025) of research on hydrological modeling approaches used to assess flood responses under LULC transitions. A total of 114 publications were retrieved from the Scopus database, and after applying PRISMA-based screening, 78 peer-reviewed studies were analyzed using bibliometric and content mapping. The review categorizes hydrological models by spatial scale, process representation, and sensitivity to LULC dynamics. Findings consistently indicate that urban expansion, deforestation, and vegetation loss intensify surface runoff, peak flow, and flood frequency. Despite advancements, significant challenges remain particularly related to data scarcity, model calibration, and the limited integration of socio-economic variables. Emerging tools such as Remote Sensing (RS), Geographic Information Systems (GIS), and machine learning especially within platforms like Google Earth Engine (GEE) enhance LULC detection accuracy and flood prediction capability. The study proposes an integrated decision framework linking bibliometric trends with model selection strategies, enabling researchers to align model choice with data availability and landscape characteristics. Overall, this review emphasizes the importance of interdisciplinary, data-driven modeling to strengthen flood resilience in rapidly transforming land systems.

## Review Highlights

• A systematic review of 78 articles (2005–2025) using PRISMA and bibliometric mapping.
• LULC changes, like urbanization and deforestation, strongly increase flood risk and runoff.
• Introduces a framework linking bibliometric findings to model selection for flood mitigation.

## Graphical abstract

**Figure:**
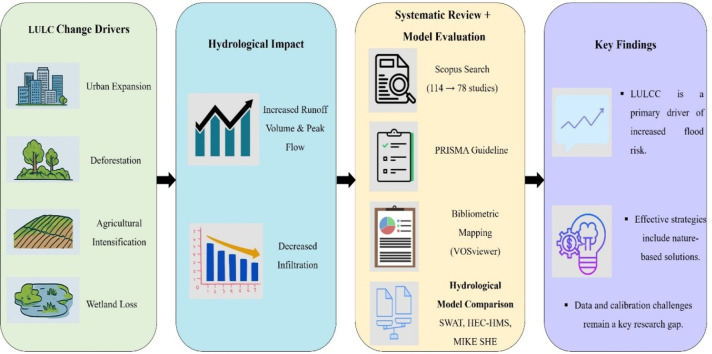

## Specifications table

**Table:** 

**Subject area**	Environmental Science
**More specific subject area**	Hydrology, LULC, Flood Modeling, RS and GIS
**Name of the reviewed methodology**	Systematic review and bibliometric mapping based on PRISMA guidelines.
**Keywords**	LULC change; Flood dynamics; Hydrological models; PRISMA guidelines; Bibliometric analysis
**Resource availability**	N.A.
**Review question**	RQ1. How do different types of LULC changes such as urbanization, deforestation, agricultural expansion, and wetland loss affect flood dynamics, including surface runoff and peak discharge? RQ2. Which hydrological models are most applied to evaluate LULC impacts, and how do their structures, data requirements, and spatial scales differ? RQ3. How have RS, GIS, and machine learning—particularly GEE—improved LULC detection and flood modeling accuracy? RQ4. What are the major challenges, data limitations, and research gaps in integrating LULC change analysis with hydrological modeling for flood risk assessment?

## Background

Rapid human development and rising living standards have profoundly transformed global land surfaces. More than 80 % of the Earth’s natural land resources have already been degraded by human activities, with the most severe impacts found in densely populated regions. Effective land management and spatial planning are essential to sustain these altered landscapes and to advance the Sustainable Development Goals (SDGs) [1]. LULC change, caused by both human activities and natural processes such as deforestation and urban expansion, affects hydrology. Combined with climate variability, these transformations alter river basin dynamics and water balance, influencing flood generation, groundwater recharge, and ecosystem stability [2].

Soil permeability, LULC changes, and topographic variation are three key watershed characteristics that control the rainfall-runoff process. Alterations in these factors reduce infiltration and storage capacity, making LULC change a major driver of urban flooding. Rapid urbanization, driven by population growth and migration to cities, remains the leading cause of land use transformation. By 2050, about 75 % of the global population is projected to live in urban areas, and the hydrological consequences of unplanned development are expected to intensify, increasing both social and environmental vulnerabilities [3].

LULC transformations directly affect key hydrological processes such as infiltration, baseflow, groundwater recharge, and surface runoff [4]. Understanding and quantifying these impacts require hydrological modeling frameworks that can link spatial land transitions to runoff responses. In data-scarce regions, lumped models are often preferred for their simplicity and low data requirements. However, advances in computing power and high-resolution RS have encouraged wider use of distributed and physically based models [5].

A wide range of hydrological models such as SWAT, HEC—HMS, MIKE SHE, TOPMODEL, and WEPP—have been developed to simulate complex water-cycle processes and evaluate the hydrological impacts of LULC change. When integrated with GIS, these models enable spatially explicit analysis of runoff generation, soil erosion, and seasonal flow variability. Their accuracy, however, largely depends on the quality of spatial datasets derived from RS, which provide consistent and up-to-date LULC information [6].

Despite extensive individual studies, few reviews have systematically integrated LULC analysis and hydrological modeling to evaluate flood dynamics within a unified analytical framework. Addressing this gap, the present review synthesizes two decades of research (2005–2025) using PRISMA guided systematic screening, bibliometric mapping, and comparative model evaluation. It identifies dominant research trends, key modeling tools, and persisting challenges in simulating LULC-driven flood processes. Unlike earlier reviews that mainly describe model applications or case studies, this work provides a broader and more integrated perspective. Specifically, it contributes threefold: (i) a reproducible bibliometric mapping of global research patterns, (ii) an evidence-based comparison of model performance and LULC sensitivity, and (iii) a transferability-oriented framework that clarifies when and how findings can be generalized across hydro-climatic and socio-environmental contexts.

## Method details

### Literature search strategy

Scopus was selected as the primary data source due to its extensive disciplinary coverage and inclusion of high-quality, peer-reviewed literature (Burnham et al., 2006; [7]). Its broad, multidisciplinary scope, spanning environmental science, engineering, and social sciences, makes it an ideal database for comprehensive systematic reviews [8]. The standardized metadata structure of Scopus also ensures consistent export formats compatible with bibliometric mapping tools such as VOSviewer and Bibliophagy. Using a single, high-quality database minimizes duplication errors and enhances the replicability of the search process. Although other databases like Web of Science or Google Scholar could potentially expand the dataset, comparative studies indicate that Scopus and Web of Science share substantial overlap in indexed journals, and their inclusion would not materially alter the overall trends identified in this review. To identify current and critical issues related to the influence of LULC changes on flood dynamics, a search strategy was developed using the keywords "LULC change," "hydrological modelling," and "surface runoff" along with their relevant synonyms (Table 2). Boolean operators (AND) were used to structure and refine the search, ensuring a focused yet comprehensive selection of relevant studies.

**Table 2:** Keywords and synonyms used for database search.

Keywords	Synonyms
LULC change	Land use change, Land cover change, Land use and Land cover change, LULC transition, Urbanization, Land degradation, Deforestation/reforestation
Hydrological modelling	MIKE SHE, WEPP, HEC—HMS, TOPMODEL, and SWAT
Surface runoff	Overland flow, Runoff, Rainfall runoff

To ensure the relevance, quality, and accessibility of the literature, the selection process was guided by explicit inclusion and exclusion criteria (Table 3). The search was narrowed to include only peer-reviewed journal articles and review papers published between 2005 and 2025. Furthermore, only English-language articles accessible through major academic databases were considered. Grey literature, dissertations, and non-English sources were excluded to maintain consistency and academic rigor. These selection criteria strengthen the credibility and relevance of the literature while ensuring the review remains focused and appropriately scoped.

**Table 3:** Inclusion and exclusion criteria for literature selection.

Criteria	Inclusion	Exclusion	Justification
Publication Year	Jan 2005 – Jun 2025	Published before 2005	Focus on the most recent and relevant decade of flooding due to LULC change effect
Document Type	Peer-reviewed journal articles, and review papers	Conference papers, book chapters, editorials, grey and literature	Ensures scientific standard and relevance for systematic analysis
Language	English	Non-English languages	Ensure consistency and align with researcher’s language proficiency
Accessibility	Open access	Subscription-based articles	Enhancing transparency, facilitates replication, and accounts for potential limitations in institutional access

**Fig. 1:**
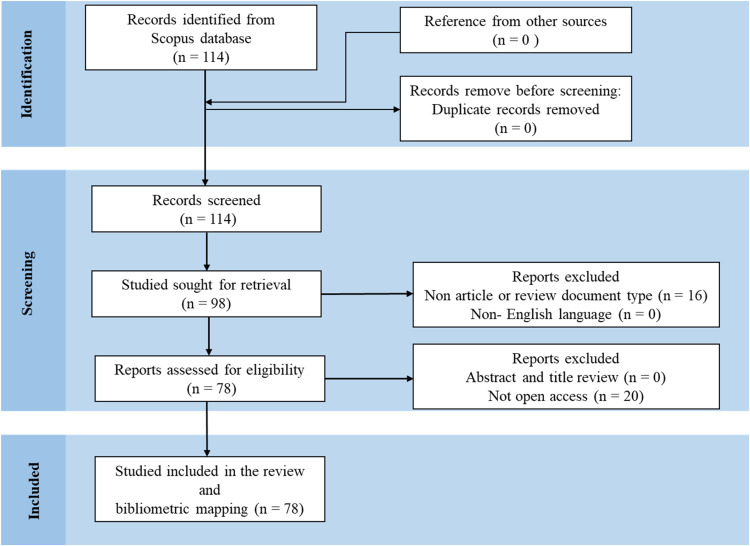
Identification of studies using PRISMA guidelines.

A total of 114 records were initially retrieved from the Scopus database, covering the period from 2005 to 2025. Following an initial screening, the selection was limited to peer-reviewed journal articles and reviews, which refined the dataset to 78 records. All non-English papers were subsequently excluded, though none were found at this stage. After applying accessibility criteria, the final dataset comprised 78 eligible articles for analysis. This systematic screening process is illustrated in Fig. 1.

**Fig. 2:**
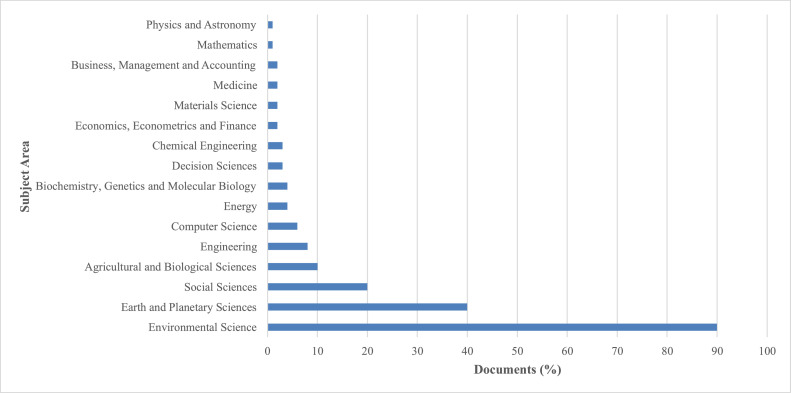
Distribution of reviewed publications by subject area in Scopus (2005–2025).

Furthermore, the retrieved data was processed using the bibliometric analysis tools VoSViewer and Bibliophagy to detect research trends, highlight influential contributors, and map collaboration and keyword networks within the reviewed literature. VoSViewer was specifically employed to identify key research trends on flooding caused by LULC changes as modeled by hydrological tools. The results were then visualized using Microsoft Excel. This analysis revealed that the subject-area distribution of the publications was dominated by Environmental Science (88 %), followed by Earth and Planetary Sciences (39 %) and Social Sciences (23 %) (Fig. 2) [9]. Fig. 2 indicates that Environmental Science and Earth Sciences dominate, reflecting the interdisciplinary scope of LULC–flood studies.

**Fig. 3:**
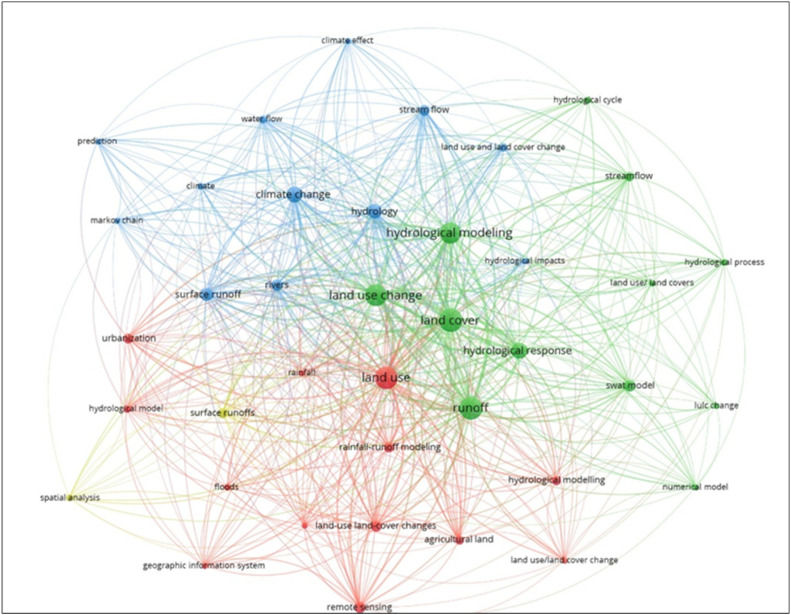
Keyword co-occurrence network of LULC-flood studies generated in VOSviewer.

The keyword co-occurrence map, illustrated in Fig. 3, reveals four distinct research clusters based on keyword frequency and collaboration. Quantitatively, hydrological modeling (56 occurrences), land use change (47), and climate change (41) were the most frequently cited keywords. The most productive journals in this domain include Water, Hydrology, and RS, collectively accounting for over 30 % of the retrieved publications. Citation trends also indicate a growing emphasis on machine learning-based flood prediction studies after 2020, reflecting an evolving methodological focus. Cluster 1 (Green) primarily focuses on "Hydrological Modeling and Response," centered on keywords such as hydrological modeling, land cover, runoff, hydrological response, and SWAT model. This cluster reflects the strong emphasis on using physically based hydrological models to simulate surface runoff, evaluate watershed responses, and assess the impacts of land surface changes. Cluster 2 (Red) addresses " RS and LULC Detection," with keywords including land use, urbanization, RS, rainfall-runoff modeling, and agricultural land. These terms represent research efforts focused on LULC classification using satellite data, GIS tools, and spatiotemporal mapping to detect land transitions that influence flood dynamics. Cluster 3 (Blue) relates to "Climate and Streamflow Interactions." This group contains terms such as climate change, streamflow, water flow, and prediction, highlighting the growing integration of climate variables and long-term hydrological forecasting in LULC-flood research, particularly for future flood risk assessment. Finally, Cluster 4 (Yellow) is defined by "Spatial Analysis and Modeling Tools," which includes keywords such as spatial analysis, hydrological model, and geographic information system. This cluster indicates a strong methodological focus on integrating spatial data and analytical platforms for modeling LULC impacts on flood behavior.

## Results

The results section synthesizes the key findings from the systematic review, beginning with a discussion of global and regional trends in LULC changes. It then outlines the technological advancements in re RS and modeling that enable monitoring of these changes, before detailing the specific hydrological impacts of LULC on runoff and flood dynamics as quantified by various hydrological models.

### Trends and patterns in LULC change

Although LULC are closely interrelated, a change in one does not always directly entail a change in the other. While land use activities often alter land cover, this does not always directly imply land degradation. Nevertheless, shifts in how land is used, often driven by socioeconomic pressures, tend to reshape the landscape. These transformations directly influence biodiversity, disrupt hydrological and energy cycles, and contribute to broader shifts in global climate dynamics [10]. LULC transitions are among the most prominent human-driven environmental changes, with significant effects on hydrological processes and flood behavior. One of the most notable trends is urbanization, particularly in developing countries, transforming rural landscapes into impervious built environments such as roads and rooftops. This transformation leads to reduced infiltration and evapotranspiration, increased surface runoff, and a disruption of the natural hydrological balance [11,12]. Agricultural expansion, which often involves clearing forests and grasslands for cropland or pasture, is another major driver of LULC shift with similar hydrological consequences.

Deforestation and forest degradation are commonly caused by agricultural expansion, logging, and plantation development, especially in tropical regions, can have serious consequences for watershed function. The loss of canopy cover reduces rainfall interception, increases soil erosion, and contributes to higher peak flows during storm events [11]. Similarly, industrial and infrastructure development, including the construction of roads, dams, and urban facilities, alters land cover and disrupts natural drainage networks, often amplifying flood risk. The conversion of wetlands into agricultural or urban areas, for example, reduces the land's ability to retain water, which limits groundwater recharge and weakens the natural regulation of floods. Conversely, reforestation and afforestation efforts in some regions, such as East Asia, represent positive land cover changes that can enhance carbon storage and hydrological stability though their benefits depend heavily on vegetation and management practices [11]. These diverse forms of LULC change underscore the need to closely examine how each transition type uniquely influences the hydrological cycle and flood behavior across spatial and temporal scales. The scale of LULC change worldwide has been significant. From 1990 to 2010, tropical forest areas shrank by approximately 121 million hectares. Southeast Asia alone experiencing a net loss of 32.96 million hectares, equivalent to an average annual decline of 6 %. These losses were primarily linked to human-driven activities, including large-scale logging, agricultural expansion, and the development of plantation-based economies (Liu et al., 2025).

**Fig. 4:**
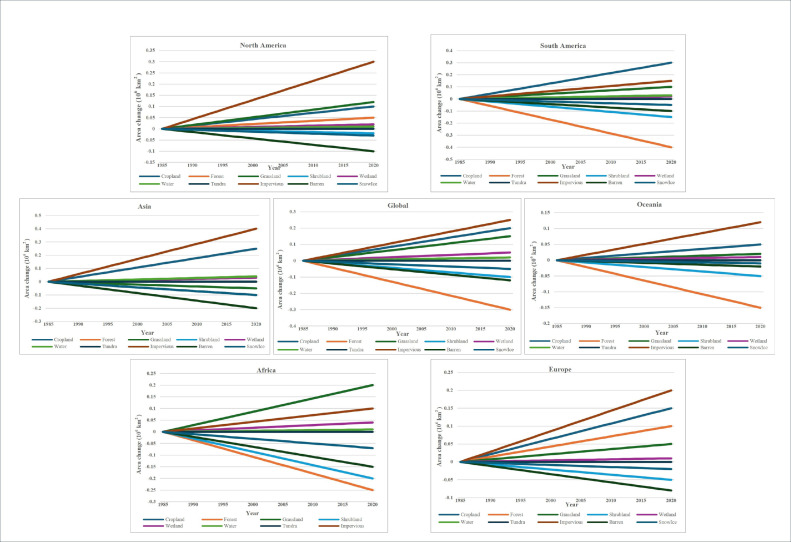
Global and regional trends in land cover change between 1985 and 2020 [9].

In the context of Thailand, a country with a globally integrated and open economy that ranked first in GDP among Indo-China Peninsula nations and eighth in Asia in 2017, several regional studies have provided insights into LULC patterns. For example, Walsh et al. [13] examined LULC and NDVI patterns in the Nang Rong region of northeastern Thailand, revealing that socio-economic drivers such as deforestation and agricultural expansion significantly influenced land transitions [13]. Wiriyanuwatkul et al. (2012) conducted a quantitative study on land use changes between 2000 and 2007, highlighting notable declines in forested areas and croplands, alongside increases in grasslands, wetlands, and urban settlements. This study found that the most extensive deforestation occurred in the northern region, while the loss of agricultural land was most pronounced within the central, eastern and southern zones of the country, with urban expansion observed nationwide. Furthermore, Wijitkosum et al. (2015) analyzed land use around the Huaxi Research Center, finding that forest regeneration played a crucial role in reducing drought severity and desertification risk. Despite these valuable insights, LULC studies in Thailand remain primarily focused on localized areas such as provinces, watersheds, and border zones. There is a notable absence of long-term, large-scale national assessments and limited application of quantitative models to analyze spatial-temporal trends and underlying drivers of change [14]. Fig. 4 illustrates significant forest loss and expansion of urban and agricultural areas across tropical regions, particularly Southeast Asia, underscoring the spatial extent of human-induced land transitions influencing flood dynamics.

### Technological approaches for monitoring and modeling LULC change

Several global land cover datasets derived from RS have been made publicly available, including DISCover (IGBP), GLC2000, MODIS MCD12, and GLCC. While widely used, these datasets' coarse resolution (approximately 1 km) and moderate accuracy (60–80 %) limit their suitability for detailed LULC analysis. GlobeLand30 offers a notable improvement with a 30-meter resolution and over 80 % classification accuracy, according to independent evaluations. However, its reliance on manual interpretation poses challenges for annual-scale updates and automation. As research and decision-making increasingly relies on up-to-date and detailed land information, there is a growing need for mapping tools that utilize high-resolution imagery, smarter classification algorithms, and automated systems that can scale effectively [14].

Rapid socioeconomic development often leads to major LULC transitions, as agricultural areas are frequently transformed into urban zones during accelerated urbanization. To better understand these spatial and temporal dynamics, researchers increasingly rely on advanced tools such RS and GIS. Numerous global datasets and computational methods have been developed to support the monitoring and analysis of evolving land use patterns [15].

Numerous automatic land classification algorithms, including Minimum Distance Classification (MDC), Maximum Likelihood Classification (MLC), Classification and Regression Trees (CART), Random Forest (RF), Support Vector Machine (SVM), Back Propagation (BP) neural networks, multiscale segmentation, and object-oriented classification methods [14]. Among these, data-driven modeling approaches like Artificial Neural Networks (ANN), Multi-Criteria Evaluation (MCE), and Logistic Regression (LR) are widely applied to estimate the likelihood of land use transitions. ANN and Cellular Automata (CA) models provide a flexible and effective framework for simulating and predicting urban growth and other dynamic changes. Given the unpredictable nature of land use change, Monte Carlo-based CA models are often employed to simulate future scenarios by assigning transition probabilities to individual cells within the modeled landscape [5].

Akar et al. (2020) evaluated the performance of RF, SVM, and MLC classification algorithms using multispectral imagery in Turkey's Trabzon region. Similarly, numerous studies have applied these algorithms across various geographic and thematic contexts. However, due to differences in model parameters, landscape characteristics, and data conditions, identifying a universally optimal classification method remains challenging. As a result, adopting a consistent classification approach such as RF - across the broader Central Asian region may offer practical advantages. RF, which integrates multiple decision trees, has gained widespread use for its robustness and classification accuracy. Although robust in handling high-dimensional datasets, RF remains underutilized in large-scale LULC studies. Traditionally, training samples have often been derived through manual visual interpretation, a method that is both labor-intensive and impractical for national or continental applications.

**Fig. 5:**
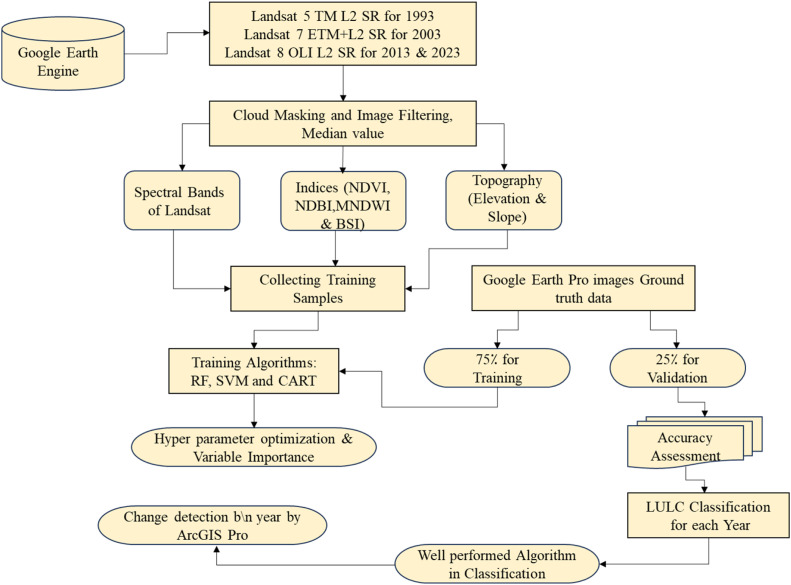
LULC classification in GEE [16].

The emergence of the GEE has significantly transformed this landscape by offering cloud-based computation capabilities for both raster and vector data. With APIs in JavaScript and Python, GEE eliminates the need to download remote sensing datasets and provides an efficient solution for scalable land cover mapping at national to global scales [14]. As an open-access platform, GEE is highly suitable for large-scale, automated LULC classification due to its large storage capacity, robust computational power, and integrated classification algorithms. A key advantage of GEE is that it eliminates the need for labor-intensive preprocessing tasks such as image mosaicking, resampling, projection transformation, and registration. Machine learning classifiers like RF, SVM, and CART on GEE are favored for their improved accuracy and classification performance (see Fig. 5) [16].

### Hydrological impacts of LULC change

LULC alterations directly affect the hydrological cycle by modifying precipitation patterns, infiltration, percolation rates, and water discharge into rivers and streams. These changes can shift water availability, increase flood risk, and reduce water quality. Beyond these direct effects, LULC transitions also influence hydrological responses indirectly by altering the landscape’s physical, chemical, and biological characteristics, including vegetation cover, soil properties, and land management practices. Such transformations disrupt natural water transport and regulation processes, further degrading water resources and ecosystem integrity. Understanding these complex relationships is crucial for sustainable water management and land use planning. The conversion of permeable land surfaces into impervious areas significantly alters hydrological behavior. It increases storm runoff, reduces vegetative interception, and accelerates flow into river systems, often resulting in water quality deterioration. The expansion of impervious surfaces through the conversion of forests, farmlands, and wetlands into urban spaces—further disrupts watershed hydrodynamics by intensifying runoff rates and diminishing groundwater recharge (Babaremu et al., 2024).

Several studies have examined the relationship between LULC change and hydrological response using different modeling approaches. Ali et al. (2011) applied the HEC—HMS model integrated with GIS to simulate how various land use scenarios affect runoff. Kalantari et al. (2014) used a distributed hydrological model to evaluate how LULC shifts influenced peak flow and runoff in six catchments, while Sayal et al. (2014) analyzed sub-catchment-scale responses to identify the effect of land use change on peak discharge. In an urban setting, Zope et al. (2015) investigated temporal LULC changes in a Mumbai watershed, emphasizing the hydrological consequences of rapid urbanization. These studies demonstrate that spatial and temporal monitoring of LULC dynamics is crucial, particularly in developing regions where land transformation is accelerating. Understanding how urban growth and LULC transitions alter surface runoff is essential for designing effective flood management and disaster mitigation strategies [17]. However, Bahremand et al. reported a 12 % decrease in peak discharge following a 50 % increase in forest cover, showing that greater vegetation improves water retention and reduces runoff intensity. This finding reinforces the profound influence of LULC change on watershed hydrology and highlights the need for continued investigation to support sustainable water resource management [18].

### Application of hydrologic models on the impacts of LULC change on runoff

Numerous studies have quantified the effects of LULC transitions on surface runoff, consistently linking urban expansion and deforestation with increased runoff volume and altered discharge regimes. Lacher et al., (2019) demonstrated that increased forest cover effectively reduces runoff volumes, while Ahmadisharaf et al. (2020) found that urban expansion, characterized by rising impervious surfaces, contributes to higher runoff volumes and elevated flood risk. Through numerical modeling, Alamdari et al. (2022) illustrated how different land use scenarios alter runoff patterns, highlighting the critical role of land use planning in water resource management. More recently, Liu et al. [19] proposed a structured framework, based on a method developed by Mayou in 2023, to evaluate the long-term effects of land use changes on watershed runoff [19].

**Fig. 6:**
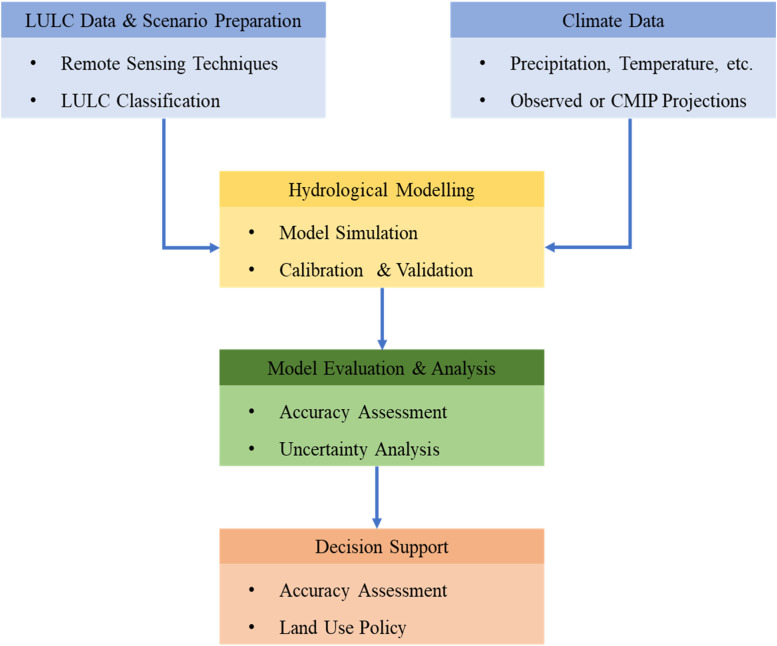
LULC-driven hydrological modeling and decision-making.

Analyzing discharge variability and hydrological responses to LULC transformations is fundamental for sustainable water management and ecological conservation within catchments. Hydrological models are widely used to examine how climate factors and human-induced land use changes affect watershed dynamics. Tools such as MIKE SHE, WEPP, HEC—HMS, TOPMODEL, and SWAT have been developed to simulate complex and spatially variable processes within the hydrological cycle. These models play an increasingly important role in supporting water resource planning and flood mitigation strategies. When combined with GIS, spatial modeling becomes a powerful method for analyzing the effects of LULC changes on runoff, sediment transport, groundwater recharge, and seasonal flow patterns. The effective implementation of these tools relies on access to comprehensive earth surface and socioeconomic datasets, with remote sensing offering an efficient and up-to-date source of LULC information for simulation and scenario analysis [6] (Fig. 6).

Table 4 provides a comprehensive summary of studies examining the impact of LULC changes on discharge. These recent findings highlight the complex ways in which land use changes affect water flow, reinforcing the need to consider these dynamics in water resource planning and management.

**Table 4:** Overview of previous investigations into the impact of LULC on runoff.

Region	Dataset	Model(s)	LULC Prediction Method	Key Outcomes	Source
Chi River Basin, Chaiyaphum Province, Northeastern Thailand	- RS & GIS data - Runoff, rainfall, socio-economic data	SCS-CN	CLUE-S with Markov Chain + Logistic Regression	- LULC scenarios for 2029–2049 under dry/normal/wet years - Runoff estimated using SCS-CN - Optimal LULC allocation identified	[20]
Songkhram River Basin, Thailand	- Land use maps (2002–2014) - Climate & sediment data - Soil map	SWAT	Dyna-CLUE (2030, 2050, 2080) under Economic & Conservation scenarios	- LULC changes had modest but positive effects on streamflow	[21]
Yom River Basin, Thailand	- Landsat 5 and 7 satellite images (1990, 1994, 1998, 2002, 2006)	MIKE 11 /NAM	No LILC forecasting was implemented in the analysis	- Increased urbanization and agricultural areas and decreased forested areas impact discharge (mean and extreme flows).	[22]
Chi River Basin, Thailand	LULC and Soil (LDD), Observed Discharge (RID)	SWAT	Based on Scenarios	- Different LULC scenarios impacted water yield and evapotranspiration seasonally and annually	(Homdee et al., 2011)
Krishna River basin, India	- Meteorological data (1980- 2013) - Climate model data (1970- 2005) - CORDEX- South Asia database - Streamflow data - Details of significant water storage structures	SWAT	CA-Markov Chain model (2025, 2055, 2085)	- LULC change, climate variability, and reservoirs significantly impacted water balance components	(Chanapathi et al., 2020)
Zhanghe River Basin, China	-World Soil Database - China Land Cover Dataset - DEM, Meteorological data - Daily runoff data	SWAT, BiLSTM	Extreme LULC method, coordinated LULC configuration method	- Urban land changes had the greatest runoff impact; models emphasized management for flood risk	[19]

## Discussion

This discussion synthesizes the influence of LULC changes on flood dynamics, examines various hydrological modeling approaches used for flood assessment, identifies the major LULC drivers of flood generation, and highlights the critical challenges and future directions in this study

### Synthesizing the influence of LULC on flood dynamics

Changes in LULC are a critical anthropogenic driver of altered flood dynamics, primarily by modifying key components of the hydrological cycle. The most direct and consistently observed consequence is an increase in surface runoff, particularly in urban areas where impervious surfaces like roads and buildings prevent water from infiltrating the soil. This leads to higher volumes that flow quickly over land [23]. Similarly, the clearing of forests and grasslands for agriculture reduces canopy interception and soil permeability, allowing stormwater to reach river systems more rapidly and increasing peak discharge during rainfall events. These transitions also diminish the natural water retention capacity of ecosystems like forests and wetlands, which in turn raises the likelihood and severity of flooding [17].

These effects underscore the significant role of LULC transitions in shaping watershed responses to precipitation and contributing to growing flood risks. Uncontrolled land use developments, often a result of inadequate zoning or rapid population growth, exacerbate this issue. Developments along riverbanks enhance the variability and severity of flood events. Therefore, understanding land use change is a crucial component of sustainable urban and environmental management, especially at the catchment scale, as it allows for the minimization of its impacts on ecosystems and hydrology. Effective land use management strategies can significantly reduce surface runoff and mitigate flood hazards. To support these strategies, accurate calculation and estimation of flood and runoff volumes are essential, providing planners and managers with the data needed for informed decision-making and proactive measures [23]. Our review confirms that these trends are a consistent finding across the literature, highlighting the need for proactive land management strategies that consider this hydrological feedback to mitigate growing flood risks.

### Modeling approaches for LULC-driven flood assessment

Assessing the hydrological impacts of LULC change requires the use of specialized hydrological models, which vary in complexity, spatial scale, and approach. Our review found that SWAT is widely used for long-term, watershed-scale simulations, making it suitable for evaluating water balance and streamflow under various LULC and climate scenarios [24]. In contrast, the HEC—HMS model is predominantly used for event-based rainfall-runoff simulations and is particularly effective for generating flood hydrographs [25]. More physically based, spatially distributed frameworks like MIKE SHE is ideal for simulating detailed surface-subsurface interactions, while empirical methods like the SCS-CN are often integrated into GIS platforms for rapid runoff estimation (Butts et.al., 2005; [26]). Other models like TOPMODEL and WEPP differ in how they represent hydrological processes and are typically chosen according to the study’s goals, data availability, and the required spatial or temporal [27]. The choice of model is typically contingent on the study's objectives, data availability, and the required spatial or temporal resolution, emphasizing a need for a tailored approach in each research context.

Recent global studies have increasingly coupled RS and machine learning techniques with process-based hydrological models to improve flood prediction accuracy and spatial scalability. For instance, Jodhani et al., [28] demonstrated the effectiveness of combining machine learning algorithms with physically based models for data-driven flood forecasting [28]. Similarly, Jodhani et al., [29] integrated RS data with artificial intelligence to quantify LULC–flood interactions under climate variability [29] while Jodhani et al., (2023) emphasized how hybrid model coupling enhances flood hazard mapping and early-warning systems (Jodhani et al., 2023). These studies highlight a growing shift toward multi-source, data-driven modeling frameworks capable of capturing nonlinear flood-land use dynamics at global scales. A comparative summary of commonly applied hydrological models for assessing flood responses under LULC change is presented in Table 4. This table outlines key model characteristics, including type, spatial scale, data requirements, LULC sensitivity, and the respective strengths and limitations of each modeling approach (Table 5).

**Table 5:** Comparative summary of hydrological models for assessing flood impacts under LULC.

Model	Type	Spatial Scale	Data Requirements	LULC Sensitivity	Key Features	Limitations
SWAT	Physically based, semi-distributed	Watershed (large scale)	High (DEM, LULC, soil, weather, land management)	High – LULC influences CN, roughness, and evapotranspiration at the HRU level; sensitive to land management and vegetation changes [24]	Long-term simulation of runoff, evapotranspiration, sediment	Complex setup requires detailed calibration
HEC—HMS	Conceptual or semi-distributed	Sub-basin to regional	Moderate (rainfall, soil, LULC, streamflow)	Medium – LULC effects modeled through CN and infiltration parameters; suitable for short-term or event-based land use scenarios [25]	Event-based simulation, flood hydrograph generation	Limited long-term simulation; simplistic LULC representation
MIKE SHE	Fully distributed, physically based	Catchment to basin scale	Very high (climate, land use, soil, groundwater)	Very High – Simulates coupled surface–subsurface flow; land cover determines infiltration, ET, and recharge; strong sensitivity to vegetation and soil properties (Butts et al., 2005)	Integrated surface-subsurface simulation, water balance modules	Intensive data input, high computational cost
SCS-CN	Empirical (lumped or GIS-based)	Plot to sub-watershed	Low to moderate (rainfall, CN values, LULC, soil)	Medium–High – CN tables directly link LULC and soil type; quick estimation of runoff under different land-use conditions but lacks seasonal variability [26].	Simple runoff estimation tool; easy GIS integration	Static assumptions; oversimplify complex processes
TOPMODEL	Conceptual, semi-distributed	Hillslope/small watershed	Moderate (topography, soil moisture, rainfall)	Medium – LULC influences saturation and contributing areas through transmissivity and soil parameters; most effective in humid catchments [27].	Captures variable source areas, topographic control	Limited representation of anthropogenic changes
WEPP	Physically based, distributed	Field to hillslope	High (climate, soil, land use, topography)	Medium – LULC affects infiltration and erosion through slope, cover, and management parameters; more accurate for field-scale assessments (Flanagan et al., 1995).	Soil erosion modeling with land management scenarios	Focus on sediment yield; less used for flood volume estimation

### Major LULC drivers of flood generation

**Fig. 7:**
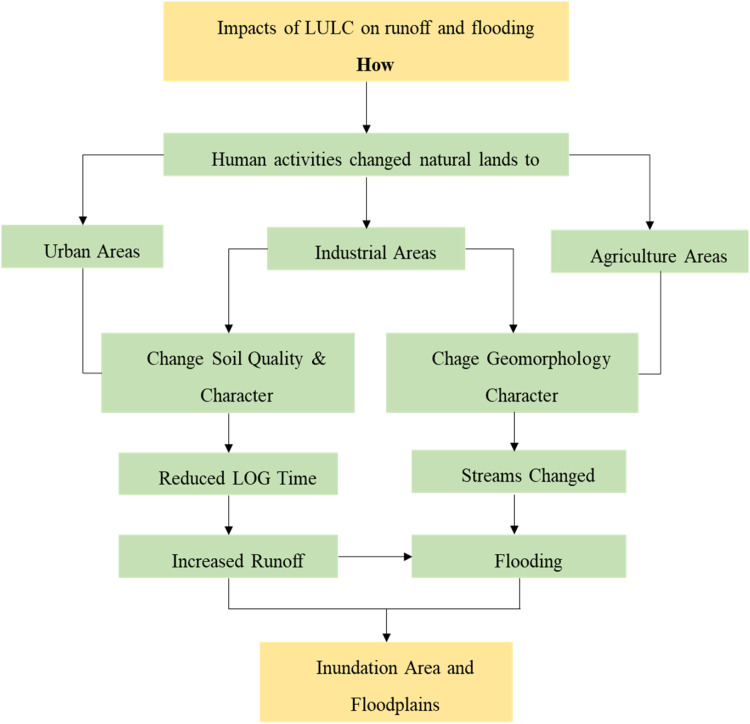
Impacts of LULC changes on runoff, urban flooding [23].

Literature consistently identifies specific LULC changes that have a particularly strong influence on flood generation. Urbanization stands out as one of the most significant drivers, as it introduces widespread impervious surfaces that drastically reduce infiltration and increase runoff. Similarly, deforestation contributes to flood risk by removing vegetation that would otherwise intercept rainfall and protect soil from erosion, thereby increasing the volume and speed of runoff [23]. The expansion of agriculture and the loss of wetlands further exacerbate flooding by altering soil structure and eliminating natural water retention areas, respectively [3]. These LULC transitions weaken the landscape’s ability to absorb and manage water, leading to more frequent and severe flood events, a trend that is particularly pronounced in rapidly developing regions. Fig. 7 illustrates the impact of LULC change on runoff generation at both urban and catchment scales.

While many of the reviewed studies are concentrated in Southeast Asia particularly Thailand because of its rapid urban growth and accessible datasets, the core hydrological processes discussed in this review are globally consistent. The connections between increased impervious surfaces, reduced infiltration, vegetation loss, and higher runoff occur in all climatic zones, though the scale of their impacts depends on local factors such as rainfall intensity, soil type, and topography [11]. Therefore, the proposed framework can be applied worldwide, if model parameters are adjusted to reflect regional hydrometeorological and land-management conditions [12]. Similar patterns of LULC-driven flooding have been observed in rapidly urbanizing parts of South Asia and Africa, deforested regions of the Amazon Basin, and intensively farmed landscapes in Europe [1]. Although Southeast Asia serves as the main empirical focus of this review, the mechanisms it highlights reflect universal hydrological responses to land use change across diverse environments.

### Transferability of findings across regions and climatic settings

Although many of the studies analyzed in this review originate from Southeast Asia, particularly Thailand, the hydrological mechanisms identified are universally applicable to other regions. The relationships between increased impervious surfaces, reduced infiltration, and higher runoff are consistent across different climates, though the degree of response varies with rainfall intensity, soil type, and topography [11]. The proposed framework can be adapted globally by recalibrating hydrological model parameters to reflect regional hydrometeorological and land-management conditions [12].

Comparable patterns have been reported in urbanizing basins of South Asia, Sub-Saharan Africa, and Latin America, as well as in agricultural and forested regions of Europe and North America [1]. This suggests that while Southeast Asia serves as a data-rich reference region, the conceptual model of LULC–flood interactions remain valid across diverse hydro-climatic settings. Integrating region-specific datasets, such as local rainfall-runoff observations or soil moisture records, would enhance the reliability and applicability of the proposed approach.

### Critical challenges and future directions

Despite the advancements in hydrological modeling and RS, several notable challenges persist in assessing flood responses to LULC change. A key issue is the limited availability of high-resolution spatial and temporal data, especially in data-scarce developing regions. Physically based models demand extensive input parameters and rigorous calibration, which can be difficult to implement. Furthermore, the inherent uncertainty in future LULC and climate projections complicates long-term flood assessments. A significant gap in current modeling approaches is the limited integration of socioeconomic factors such as land use policies and management practices, which are crucial drivers of real-world LULC dynamics. Finally, mismatches in scale between hydrological processes and available data resolution can compromise model accuracy. These challenges underscore the need for more flexible, data-driven models, better integration of diverse data sources, and interdisciplinary research to enhance flood resilience in the face of dynamic land use transitions. Future studies should explore hybrid modeling approaches that couple physically based hydrological models with machine learning techniques to improve predictive accuracy and adaptability under changing land use and climate conditions.

## Conclusion

This review highlights how changes in LULC significantly affect flood behavior and watershed hydrology. Understanding these interactions is critical for improving water resource management and flood mitigation strategies. The findings consistently show that transitions such as urbanization, deforestation, agricultural expansion, and wetland loss tend to increase surface runoff, peak discharge, and flood frequency by disrupting natural infiltration and water retention functions. Hydrological models are essential for analyzing and predicting these impacts.

The novel contribution of this review is the integration of systematic analysis, bibliometric mapping, and model comparison within one framework. It not only summarizes past studies but also connects LULC drivers with hydrological model selection. This approach helps researchers identify which models are most suitable under different data, scale, and landscape conditions. Furthermore, the study proposes a transferability-oriented framework that links bibliometric insights with model selection strategies, enabling researchers and practitioners to align hydrological model choice with local landscape characteristics, data availability, and study objectives. This integrative perspective not only clarifies where each model performs best but also identifies knowledge gaps related to data resolution, model calibration, and socio-hydrological integration.

Addressing these gaps will require interdisciplinary approaches that couple RS, GIS and scenario-based modeling with socio-environmental datasets to improve prediction accuracy and policy relevance. Advanced this research will strengthen the capacity of hydrological modeling to support adaptive, data-driven flood risk management under accelerating LULC changes and climate variability.

## Ethics statements

No data was used in this research.

## Funding

No Funding was used in this research.

## CRediT authorship contribution statement

**Tin Zar Oo:** Conceptualization, Methodology, Formal analysis, Investigation, Visualization, Writing – original draft, Writing – review & editing. **Usa Wannasingha Humphries:** Supervision, Writing – review & editing, Project administration.

## Declaration of competing interest

The authors declare that they have no known competing financial interests or personal relationships that could have appeared to influence the work reported in this paper.
